# Biological Fuel Cells and Membranes

**DOI:** 10.3390/membranes7010003

**Published:** 2017-01-17

**Authors:** Zahra Ghassemi, Gymama Slaughter

**Affiliations:** Bioelectronics Laboratory, Department of Computer Science and Electrical Engineering, University of Maryland Baltimore County, 1000 Hilltop Circle, Baltimore, MD 21250, USA; ghzahra1@umbc.edu

**Keywords:** biofuel cells, microbial fuel cells, semi-permeable membrane, chitosan, Nafion

## Abstract

Biofuel cells have been widely used to generate bioelectricity. Early biofuel cells employ a semi-permeable membrane to separate the anodic and cathodic compartments. The impact of different membrane materials and compositions has also been explored. Some membrane materials are employed strictly as membrane separators, while some have gained significant attention in the immobilization of enzymes or microorganisms within or behind the membrane at the electrode surface. The membrane material affects the transfer rate of the chemical species (e.g., fuel, oxygen molecules, and products) involved in the chemical reaction, which in turn has an impact on the performance of the biofuel cell. For enzymatic biofuel cells, Nafion, modified Nafion, and chitosan membranes have been used widely and continue to hold great promise in the long-term stability of enzymes and microorganisms encapsulated within them. This article provides a review of the most widely used membrane materials in the development of enzymatic and microbial biofuel cells.

## 1. Introduction

A conventional fuel cell is an electrochemical power source that continuously converts the stored chemical energy in a fuel to electrical energy as long as there is a continuous supply of fuel. These fuel cells consist of the fuel, oxidant, and the anodic and cathodic substrate materials. The anodic and cathodic substrates are typically separated by a semi-permeable membrane. Michael Cresse Potter in 1911 conceptualized and described a biofuel cell consisting of two platinum electrodes in the presence of *E. coli*, where a potential difference was observed between the two platinum electrodes. Since then, different biofuel cells have been developed and can be categorized as follows [1]:

1. A primary fuel is used by a biofuel cell and generates a material such as hydrogen, which can be used as a secondary fuel within a conventional hydrogen/oxygen fuel cell.
2. An organic fuel, such as glucose, is used in a biofuel cell and directly generates bioelectricity. This biofuel cell may contain enzymes or microorganisms.
3. Photochemically active systems and biological moieties are used to harvest energy from sunlight and convert it to electrical energy.

However, biofuel cells differ slightly from conventional fuel cells, in that they employ naturally occurring proteins or microorganisms as the biocatalysts for the anodic and cathodic substrate materials to catalyze the electrochemical reactions between the fuel, oxidant, and the biocatalysts. Biocatalysts such as enzymes have high electrocatalytic activity at moderate conditions (pH and temperature). In addition, they are renewable and can be developed to oxidize many different fuels. These characteristics render biocatalysts as attractive alternatives to metal catalysts employed in conventional fuel cells. Herein, we explore the study of different membranes, which have been used in organic biofuel cells employing both enzymatic and microbial fuel cells.

## 2. Enzymatic Biofuel Cell

Enzyme-based fuel cells (enzymatic biofuel cells) and microorganism-based biofuel cells (microbial biofuel cells) generate bioelectricity through the oxidation of renewable energy sources such as organic acids and sugars, coupled with the reduction of oxygen to water [2,3,4,5,6,7,8,9], and are safer than Li-ion batteries and direct methanol fuel cells [10]. These biofuel cells are expected to produce higher energy density and enable a wide range of applications, thereby becoming the next generation energy devices. Although enzymes and microorganisms are highly efficient biocatalysts, some challenges exist when it comes to the immobilization of the biocatalysts on the electrode surface. Hence, the early enzymatic and microbial biofuel cells utilized solution-borne enzymes or microorganisms rather than immobilized biocatalysts on the electrode surface. These biofuel cells were found to be stable for only a few days, whereas biofuel cells with immobilized enzymes or microorganisms were stable for several months. Heineman et al. employed a transparent thin layer electrode with a mediator to determine enzyme activity and solution potential, E°’, values using a spectropotentiostatic method [11]. The major drawback of this work is the lack of a semi-permeable membrane to separate the half-cell reactions occurring at the anode and cathode of the biofuel cell. The incorporation of a membrane material in this system is essential in order to control the enzyme and substrate reaction at the surface of the electrode. Given that a semi-permeable membrane was not employed to separate the anodic and cathodic compartments, it was very challenging for Heinemann et al. to determine the correlation between solution potential and enzyme activity. One of the earliest glucose biofuel cells that incorporated a semi-permeable membrane enabled the realization of a compact construction of the biofuel cell to enable effective mass transport and the determination of the electrode characteristics. The active anode was prepared from platinum-nickel alloy, and activated carbon or porous silver was used as the cathode material. Rao et al. [12] described the use of cuprophane, dialysis-tubing material, and foil derived from sulfonated Teflon as the semi-permeable membrane in the development of a circular biofuel cell. A stable bonding method was employed to keep all the components of the biofuel cell tightly together and the various layers of the complete circular biofuel cell was confirmed via scanning electron microscopy.

**Figure 1:**
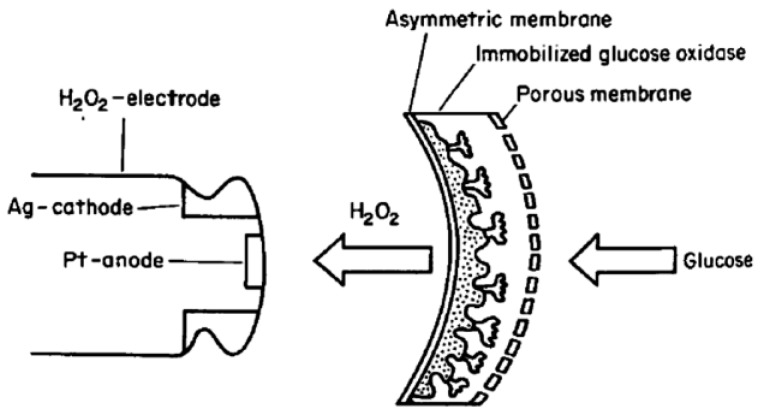
Scheme of the immobilized glucose oxidase membrane electrode.

It has become common practice to employ semi-permeable membrane material for the immobilization or physical entrapment of enzymes or microorganisms on the surface of the electrode, while separating the anodic and cathodic compartments. The semi-permeable membrane plays an integral role in the performance of a biofuel cell due to its impact on the transfer rate of different chemical species in the electrolyte/solutions. Additionally, the power density of an implantable biofuel cell as a power supply for bioelectronic devices can be significantly decreased by orders of magnitude due to the limitations to the mass transfer rate of fuels (organic acids and sugar), oxygen, and permeability barriers of the immobilized biocatalysts [13]. Tsuchida et al. employed an asymmetric membrane for the immobilization of glucose oxidase in an enzymatic biofuel cell. It was reported that the asymmetric membrane was perm-selective to hydrogen peroxide byproduct generated as a result of glucose oxidation. This perm-selective asymmetric membrane was prepared by casting acetyl cellulose in a mixed solvent of acetone and cyclohexanone, upon which glucose oxidase was immobilized. A final layer of a porous polycarbonate membrane was used to coat the electrode. Figure 1 shows the scheme of the immobilized glucose oxidase asymmetric membrane electrode. This work demonstrated that in the presence of likely interfering oxidative species, the asymmetric membrane was selectively permeable [14]. The permeability of asymmetric acetyl cellulose membrane to hydrogen peroxide was shown to increase in alkaline conditions [15].

**Figure 2:**
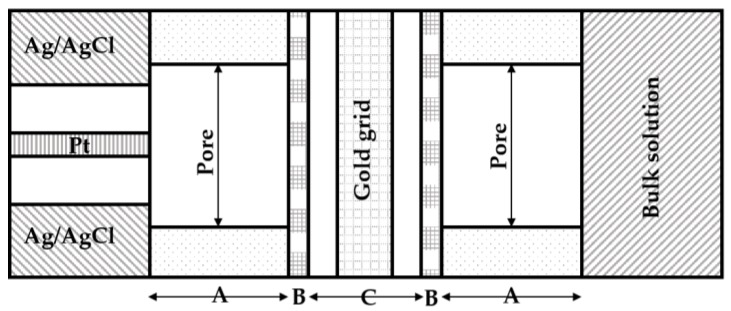
Microscopic cross-section of the permeable thin layer cell. The membrane control electrode assembly is comprised of three unique layers: polycarbonate membrane (A); cellulose acetate membrane (B); and the enzyme thin layer containing the gold grid and entrapped galactose oxidase (C).

Moreover, Johnson et al. encapsulated the working electrode with a cation exchange membrane in order to design a unique membrane-controlled electrode assembly [16]. This way, the larger cation and anion species would be preserved to perform the electrochemical experiments. A thin semi-permeable membrane was also used to separate the external bulk solution in the biofuel cell and at the electrode, as shown in Figure 2. The thin layer contained galactose oxidase as the biocatalyst and ferricyanide as the mediator. Galactose was used as the substrate fuel in this system. A gold mini-grid electrode was then sandwiched between two cellulose acetate membranes (B), and these layers were supported by a porous polycarbonate membrane (A). In addition, the gold mini-grid electrode showed rapid total electrolysis of the chemical species that were too large to diffuse out of membrane (B), whereas smaller species were able to pass rapidly through the membrane (B). The solution potential activity of substrate reactions was acquired via cyclic voltammetry. Since then, significant progress towards a membraneless biofuel cell has been made. The first membraneless bioanode was reported by Persson et al., who constructed a glucose biofuel cell with glucose dehydrogenase immobilized on the surface of a graphite felt anode [17]. A simulated oxygen electrode that generated a current-independent voltage of +560 mV versus saturated calomel electrode (SCE) was used as the cathode material. A relatively high current density (up to 10 mA·cm^−2^) was demonstrated, and this was attributed to the adsorption property of the graphite felt electrodes.

Polymers with distinct hydrophilic and hydrophobic domains can naturally form polymers with micellar structure, which are more stable than surfactant micelles [18,19,20]. Nafion is a micellar polymeric membrane that has been widely used as a membrane separator in the development of fuel cells because of its micellar porous structure, which further facilitates the transport and preconcentration of cations within the membrane [21]. Although Nafion has garnered significant attention for the immobilization of biocatalysts at the electrode surface [22], it has been shown to be quite ineffective in extending the stability of the immobilized biocatalysts. This is because of Nafion’s tendency to form an acidic membrane, which decreases the lifetime and activity of immobilized biocatalysts [23]. Therefore, a mixture cast of Nafion and quaternary ammonium salts such as tetrabutylammonium bromide have been used to improve the mass transport of small analytes through the membrane and enhance the stability of immobilized biocatalysts. In addition, Nafion has been shown to decrease the selectivity of the membrane against anions [24]. Nafion modified with tetrabutylammonium bromide and unmodified Nafion exhibit similar properties regarding cation transport properties; however, modified Nafion has been shown to exhibit higher selectivity to proton transport because the unmodified and modified versions have different acidic or basic properties. Titrations of tetrabutylammonium bromide Nafion membrane showed that the membrane resists re-exchange of protons, and this property helps buffer the pH environment, which would prevent the membrane from becoming too acidic for the biocatalysts, such as enzymes [25]. To ensure reproducible and stable modification of Nafion, the excess bromide salt that may be trapped in the pores or deactivated in the equilibrated membrane is removed from the casting solution using salt-extracted membrane. However, modified Nafion has several disadvantages; it is expensive, non-biodegradable, and not biocompatible [26].

In the search for an alternative to modified Nafion, researchers began to explore the hydrophobicity of modified chitosan [19,27]. Chitosan is formed from chitin, which is abundant in the outer shells of crustaceans and insects. It is a linear polysaccharide, and it is an inexpensive biopolymer [19,27]. Because of its biocompatibility, biodegradability, high-mechanical strength, and non-toxic properties, it has gained significant attention in the construction of biofuel cells. Hydrophobically-modified chitosan alters the flux of redox species to the electrode surface. It supplies the optimal microenvironment for enzyme immobilization [21]. The ion-exchange capacity of hydrophobically-modified chitosan and Nafion membranes were characterized [21], where the hydrophobically-modified Nafion was made by mixture casting large hydrophobic ammonium salts. The process exchanges the ammonium cations for the protons on the sulfuric acid groups on Nafion [28]. Hydrophobically-modified chitosan was made by a reductive amination with long alkyl chain aldehydes [21]. The number of exchange sites available to protons increase with hydrophobic modification. The activity of enzymes was studied using glucose oxidase immobilized within these two membrane motifs. The modified Nafion membrane modified with trimethyldodecylammonium bromide (TMDDA) suspended in ethanol and hexyl modified chitosan suspended in t-amylalcohol showed the highest enzyme activities by creating a microenvironment that stabilizes the enzymes. A two-fold increase in the enzyme activity was observed for both modified Nafion and chitosan. Accordingly, hydrophobically-modified chitosan and Nafion were observed to yield a better microenvironment for enzyme in comparison to physiological buffers. This is because the hydrophobically-modified chitosan with long chain aldehydes forms a micellar polymer which exhibits similar properties to hydrophobically-modified Nafion. In addition, the modified chitosan membrane allows for the tailoring of ion-exchange and mass transfer capacities. Minteer et al. characterized a hydrophobically-modified chitosan and Nafion membrane for the immobilization of dehydrogenase enzymes [29], where it was observed that the hydrophobically-modified chitosan membrane showed higher electrochemical flux compared to hydrophobically-modified Nafion membrane. It is important to note that the performance of a biofuel cell depends on the membrane properties, such as transport properties and the ability to extract or preconcentrate NAD^+^, in addition to the size and catalytic activity of the enzymes. Table 1 provides a summary of semi-permeable membranes commonly employed in enzymatic biofuel cells.

## 3. Microbial Fuel Cell

**Figure 3:**
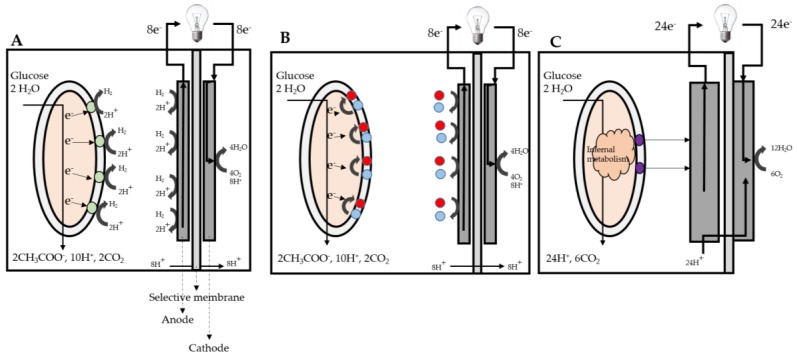
(**a**) An indirect microbial biofuel cell; (**b**) A mediator-driven microbial biofuel cell; (**c**) A direct electron transfer microbial biofuel cell.

An early example of a microbial fuel cell (MFC) was demonstrated by Potter et al., wherein cultures of *Saccharomyces cerevisiae* were grown in nutrient-rich media, and a porous cylinder was used to separate the platinum anode and cathode electrodes [34,35]. Due to the close ecological cycle of microbial fuel cells and human food, in the 1960s, Austin characterized the direct electron transfer microbial fuel cell [36]. Suzuki et al. characterized the indirect electron transfer in a microbial fuel cell using *Clostridium butyricum* [37]. In 1964, Berk et al. studied the interaction between photosynthetic microorganisms and platinum electrodes, while employing a dialysis membrane to allow for the movement of ions between the half cells and generate electricity [38]. The experiments performed by Potter et al. and Berk et al. focused on the basic principles of microbial fuel cells’ operation. The anode in a microbial fuel cell accepts electrons from the microbial culture, and the cathode transfers electrons to an electron acceptor. The cathodic compartment is typically exposed to air or suspended in aerobic solutions. However, the anodic compartment is usually kept under anoxic conditions. Through an external electrical circuit, the electrons flow from the anode to cathode, thereby generating bioelectricity. The anode and cathode are usually separated by a semipermeable membrane. This membrane prevents oxygen diffusion from the cathode chamber to the anode chamber, while allowing protons to move from the anode chamber to the cathode chamber. At the cathodic compartment, oxygen, protons, and electrons recombine to form water [39]. Figure 3 depicts the three mechanisms of electron transfer from the anode to the cathode that are employed in microbial fuel cells to produce bioelectricity.

Nafion is a widely applied semi-permeable membrane in microbial fuel cells [40,41,42,43,44,45,46,47,48]. However, the use of Nafion as proton exchange membrane (PEM) has been associated with operational challenges. For example, Gil et al. observed a decreasing pH in the anodic compartment and an increasing pH in the cathodic compartment in a two-chamber MFC because the proton production rate at the anode and proton consumption rate at the cathode were much faster than proton transport through the Nafion PEM [41]. Liu el al. [46] were able to generate bioelectricity using an air-cathode single chamber microbial fuel cell in the presence and absence of a polymeric proton exchange membrane. This arrangement was found to be effective in increasing the total energy output and at the same time reducing the cost of the development of the microbial fuel cell. Interestingly, the bacteria formed in domestic water waste were employed as biocatalysts in a MFC. Glucose and waterwaste were used as the substrate fuel and Nafion as polymeric PEM [46]. The anode was composed of Toray carbon paper and the cathode was carbon electrode/PEM cathode (CE-PEM). The CE-PEM cathode was constructed by bonding the PEM directly onto a flexible carbon-cloth electrode. In the absence of the PEM membrane, the maximum power density increased, and the Coulombic efficiency was observed to be much higher than in the presence of PEM. This indicates that substantial oxygen diffusion into the anodic compartment was occurring in the absence of the PEM. A higher power density of 146 ± 8 mW/m^2^ was observed for the glucose and waterwaste fuel in the absence of PEM. However, omitting PEM resulted in the deactivation of the platinum cathode due to the resulting contamination in the anodic compartment.

**Figure 4:**
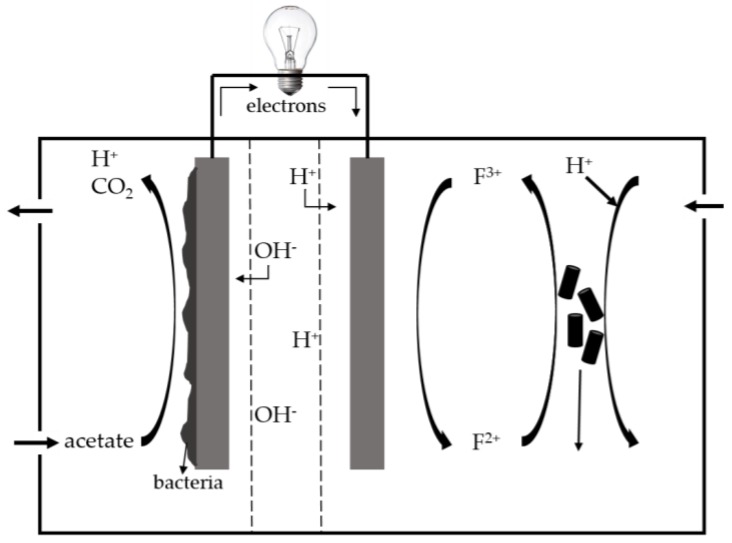
Design for the microbial fuel cell (MFC) with iron mediated cathode and bipolar membrane.

Rozendal et al. [49] investigated the effects of membrane cation transport on pH-sensitive MFC performance. MFCs that employ a Nafion PEM have the capability to transport protons and other cation species such as Na^+^, K^+^, and Mg^2+^. Cation species other than protons are responsible for the transport of positive charge through the Nafion membrane, since the concentration of these cation species are 10^5^ times greater than the protons. Thus, the cation species accumulated and led to an increase in conductivity. The protons were consumed at the cathode; thus, the transport of other cation species other than protons resulted in a decrease in the MFC performance and an increase in the cathodic pH [49]. This rise in pH resulted in an extensive iron precipitation, which eventually caused the membrane to break down. Ter Heijne et al. [50] employed a bipolar membrane with low catholyte (pH < 2.5) in the development of an MFC that employed a bipolar membrane with low catholyte less than pH of 2.5. The bipolar membrane exhibited cation and anion exchange sections, which were joined together in series [51,52]. Figure 4 shows the MFC incorporating the bipolar membrane with cathodic ferric iron reduction and the regeneration of ferric iron. *Acidithiobacillus ferrooxidans* was employed as the microorganism [50]. The MFC maintained low catholyte pH, which was required to keep ferric iron soluble and from potentially damaging the membrane. Additionally, as a result of water disassociation, the bipolar membrane provided the anodic and cathodic compartments with hydroxides and protons, respectively. No loss of iron was observed when bipolar membrane was employed.

Kim et al. [53] investigated the power densities, Coulombic efficiencies, and permeability to oxygen and a substrate, for Nafion, cation exchange membrane (CEM), anion exchange membrane (AEM), and three ultrafiltration (UF) membranes using two-chamber MFCs with different architectures. CMI-7000 (polymer structure: Gel polystyrene cross linked with divinylbenzene, functional group: sulphonic acid) was used as CEM and AMI-7001 (polymeric structure: gel polystyrene cross linked with divinylbenzene, functional group: quaternary ammonium) was used as AEM. AEM was found to produce the highest power density of up to 610 mW/m^2^ and Columbic efficiency of 72%. AEM also showed an increased performance due to the facilitation of proton exchange transfer with the ammonium cation of the AEM (–NH_3_^+^ functional groups) versus those of the PEM and CEM) with phosphate anions (–SO_3_^−^ functional groups), which exhibited low internal resistance. The UF membranes produced very high resistances and showed the least permeability to oxygen and acetate. UF membranes also showed higher permeability to acetate, whereas Nafion showed the highest permeability to oxygen [53]. Sun et al. [54] studied the performance of an air-cathode single compartment MFC for waterwaste treatment using microfiltration membranes (MFM) and multiple sludge inoculation. One layer of MFM was applied only on the water-facing side of the air cathode to improve its performance. A substantial reduction in the internal resistance from 672 to 248 was observed when PEM was exchanged with MFM, which resulted in approximately a two-fold increase in the maximum power density of MFC with MFM in comparison to MFC with PEM. The Coulombic efficiency increased from 4.17% to 5.16% when MFM was employed in place of a membraneless system. This was attributed to the chemical oxygen demand (COD) removal efficiency by using MFM [54]. Ghasemi et al. fabricated two different composite membranes that were used in MFCs, and compared their characteristics with Nafion 117 and Nafion 112. They fabricated carbon nanofiber (CNF)/Nafion and activated carbon nanofiber (ACNF)/Nafion membranes. The nanocomposite membranes were observed to exhibit higher Coulombic efficiency and production power. The MFC which had an ACNF/Nafion membrane resulted in the production of the highest power density (57.64 mW/m^2^), while the MFC employing Nafion 112 resulted in the least power density (13.99 mW/m^2^). Furthermore, as the nanocomposite membrane pore size and roughness decreases, the transfer of oxygen from the cathode compartment to the anode compartment was inhibited, and thereby prevented the migration of bacteria from the anode compartment to the cathode compartment. Additionally, the reduced roughness decreased the biofouling in the membrane while enhancing its conductivity [55]. Lim et al. [56] synthesized a PEM composite by combining sulfonated poly(ether ether ketone) (SPEEK) in poly(ether sulfone) (PES) for fabrication of a MFC. When a small amount of hydrophilic SPEEK (approximately 3%–5%) was added, the conductivity of the hydrophobic PES membrane increased. During MFC operation, the conductivity and capacitance of the PES/SPEEK composite membrane decreased. The PES/SPEEK 5% membrane exhibited the highest power density (170 mW/m^2^). The MFC with composite membrane PES/SPEEK 5% COD removal efficiency increased by 26-fold, and was two-fold higher than that of the MFC incorporating Nafion 112 and Nafion 117 membranes, respectively [56].

Current work by Kim et al. demonstrated a new method to reduce the resistance of the membrane used in MFCs. Membrane resistance is the result of low electrolyte accessibility onto the MFC membrane surface. Kim et al. [57] coated an ultrafiltration membrane with polydopamine (PD) to reduce the resistance of the membrane and create a negatively charged surface. Electrochemical impedance spectroscopy showed a significant reduction in the resistance of the UF membrane and an increase in the maximum power density. The PD-coated UF membrane resulted in a high power density (159 mW/m^2^), whereas the untreated UF membrane produced a power density of 137.9 mW/m^2^—approximately 15% less than the power density achieved by the previous PD-coated UF [57]. Recently, Hernández-Fernández et al. [58] employed a newly fabricated ionic liquids membrane as the PEM in a MFC for waterwaste treatment. The supported ionic liquid membranes comprised of 1-*n*-alkyl-3-methylimidazolium (*n*-butyl, *n*-octyl) and methyl trioctylammonium cations combined with hexafluorophosphate, tetrafluoroborate, chloride, and bis{(trifluoromethyl)sulfonyl}imide anions. Nafion and Ultrex were used as reference membranes [58]. The fabrication of the supported ionic liquid membranes was attained by transporting the corresponding ionic liquid through a Nylon membrane using Amicon UF as described in Hernández-Fernández et al. [49]. Table 2 shows the comparison of the reduction in COD (initial COD 1174 mg/L), the Coulombic efficiency, and the maximum power density achieved with different ionic liquid membranes with Nafion and Ultrex membrane serving as reference membranes [59].

The need for hydration and the high cost associated with CEMs [60] in air-cathode-based MFCs makes CEMs unsuitable for large-scale applications. Therefore, inexpensive membranes such as nylon filter, glass fiber mat, and non-woven cloth have been reported [61]. Pasternak et al. [62] compared the performance of different kinds of ceramic membranes (alumina, earthenware, mullite, and pyrophyllite), and evaluated their characteristics in a cascade of MFCs. Pyrophyllite yielded the best performance for the MFCs and achieved a power density of 6.93 mW/m^2^ [62], wherein the chemical properties of the ceramic membranes affect the cell performance.

## 4. Conclusions

Enzymatic and microbial biofuel cells have gained significant attention as a power source in biological environments, where semi-permeable membranes have been applied for the separation of anodic and cathodic compartments as well as the immobilization of enzyme in enzymatic biofuel cells. Nafion, modified Nafion, and chitosan have been widely used as semi-permeable membranes and have resulted in high power outputs. Biofuel cells employing hydrophobically-modified chitosan as semi-permeable membrane and for the immobilization of biocatalysts result in the generation of higher power density. For microbial biofuel cells, proton exchange membranes (Nafion) have continued to gain attention, and CEM and AEM have been used to improve the characteristics of microbial biofuel cells. Microbial biofuel cells which employ poly(ether sulfone) (modified PEM) have better performance than previous microbial biofuel cells. However, ionic liquid membranes comprised of 1-*n*-alkyl-3-methylimidazolium (*n*-butyl, *n*-octyl) and methyl trioctylammonium cation combined with hexafluorophosphate, tetrafluoroborate, chloride, and bis{(trifluoromethyl)sulfonyl}imide anions have resulted in an overall higher power density.

## Figures and Tables

**Table 1:** Summary of the performance of enzymatic biofuel cells employing semi-permeable membranes.

Anode	Cathode	Membrane	Fuel	Power Output	Ref.
Ag/glucose oxidase (GOX)	Pt/peroxidase	Asymmetric acetyl cellulose	Glucose/H_2_O_2_	–	[14]
Gold/galactose oxidase	Pt	Cellulose acetate and porous polycarbonate	Galactose	–	[16]
Graphite Felt/glucose dehydrogenase (GDH)	Simulated oxygen	d-3-hydroxybutyrate dehydrogenase (BDH)	Glucose	–	[17]
Au/glucose oxidase (GOX)	Au/microperoxidase	Glass frit	Glucose/H_2_O_2_	32 µW at 0.31 V vs. SCE or SCE	[30]
Au/GOX	Au/microperoxidase	H_2_O/CH_2_Cl_2_ interface	Glucose/cumene peroxide	520 µW at 1 V vs. SCE	[31]
Graphite (formate/aldehyde/alcohol dehydrogenases soln.)	Pt	Nafion	MeOH/O_2_	670 µW·cm^−2^ at 0.49 V vs. SCE	[9]
Pt	C or Pt with laccase in solution	Nafion	H2/O_2_	42 µW·cm^−2^ at 0.61 V vs. SCE	[32]
Porous C/C nanotube/GOX	Porous C/C nanotube/laccase	Nafion	Glucose/O_2_	99.8 µW·cm^−2^	[2,33]
Carbon felt/Nafion NBu_4_^+^ salt alcohol + aldehyde dehydrogenase	Pt/C	Tetrabutylammonium bromide/Nafion	MeOH/O_2_, EtOH/O_2_	1550 µW·cm^−2^, 2040 µW·cm^−2^	[25]
Carbon/GDH	ELAT^®^ (Woven carbon cloth gas diffusion layer with a carbon microporous layer)	Butyl-Chitosan	Glucose/NAD^+^	35 µW·cm^−2^, 0.699 v open circuit potential	[29]
Carbon/GDH	ELAT^®^	Octyl-Chitosan	Glucose/NAD^+^	17 µW·cm^−2^, 0.628 v open circuit potential	[29]

**Table 2:** Comparison of MFC with different ionic liquids membranes and Nafion and Ultrex membrane as reference membranes, where COD, [MTOA^+^][Cl^−^], 1-[omim^+^][NTf_2_^−^], [omim^+^][BF_4_^−^], and [omim^+^][PF_6_^−^] are chemical oxygen demand, methyl trioctyl ammonium chloride, Octyl-3-methylimidazolium bis{(trifluoromethyl)sulfonyl}imide, 1-Octyl-3-methylimidazolium tetrafluoroborate, and 1-Octyl-3-methylimidazolium hexafluorophosphate, respectively.

Membrane	COD Removal (%)	Coulombic Efficiency (%)	Maximum Power (mW/m^2^)
Nafion^®^	90.7	4.44	157.9
Ultrex^®^	88.3	2.50	102.2
[MTOA^+^][Cl^−^]	89.1	2.06	103.9
[omim^+^][NTf_2_^−^]	81.3	2.74	72.1
[omim^+^][BF_4_^−^]	80.3	1.31	147.1
[omim^+^][PF_6_^−^]	27.3	18.60	215.0
